# Uncovering the Impact of Center of Rotation of Angulation Location on High Tibial Osteotomy in Knee Osteoarthritis: A Potential Pathway for Improved Outcomes

**DOI:** 10.1177/19476035261420279

**Published:** 2026-02-18

**Authors:** Yannick Janssen, H. Chien Nguyen, Roel J. H. Custers, Nienke van Egmond, Moyo C. Kruyt, Ralph J. B. Sakkers, Jaap Thooft, Margreet Kloppenburg, Francisco J. Blanco, Ida K. Haugen, Francis Berenbaum, Simon C. Mastbergen, Harrie Weinans, Eva A. Bax

**Affiliations:** 1 Department of Orthopaedic Surgery, University Medical Centre Utrecht, Utrecht, The Netherlands; 2 3D Lab, University Medical Centre Utrecht, Utrecht, The Netherlands; 3 Departments of Rheumatology and Clinical Epidemiology, Leiden University Medical Center, Leiden, The Netherlands; 4 Grupo de Investigación de Reumatologia (GIR), INIBIC-Complejo Hospitalario Universitario de A Coruña, Centro Interdisciplinar de Quimica y Biologia, CICA-UDC, Universidad de A Coruña, A Coruña, Spain; 5 Center for Treatment of Rheumatic and Musculoskeletal Diseases (REMEDY), Diakonhjemmet Hospital, Oslo, Norway; 6 Department of Rheumatology, Sorbonne University, INSERM CRSA, AP-HP Saint-Antoine Hospital, Paris, France; 7 Department of Rheumatology & Clinical Immunology, University Medical Center Utrecht, Utrecht, The Netherlands; 8 Department Biomechanical Engineering, Faculty of Mechanical Engineering, TU Delft, Delft, The Netherlands; Investigation performed at University Medical Center Utrecht, The Netherlands

**Keywords:** CORA, lower limb malalignment, osteoarthritis, osteotomy

## Abstract

**Objective:**

Lower limb malalignment accelerates the progression of knee osteoarthritis (KOA). Knee realignment osteotomy is a well-established treatment for unicompartmental KOA with malalignment. Traditional planning in KOA patients corrects deformities with an osteotomy at the metaphysis but overlooks Paley’s approach, which targets the center of rotation angulation (CORA). Osteotomy at the metaphysis may induce secondary translational deformities, which remain unstudied in KOA patients. This study aims to identify the CORA in KOA patients with tibial malalignment.

**Methods:**

Thirty tibiae (10 varus, 10 neutral, 10 valgus) from the IMI-APPROACH cohort were analyzed using computed tomography (CT) scans. The CORA, defined as the intersection of the proximal and distal mechanical axes, was identified. Translational deformity was calculated by multiplying the CORA-to-osteotomy distance by the tangent of the correction angle.

**Results:**

Among the varus tibiae, 9 out of 10 CORAs were located in the diaphysis, while 8 out of 10 valgus tibiae had their CORA in the diaphysis. When osteotomies were performed in the proximal metaphysis instead of the CORA location, secondary translational deformities of up to 3 cm were induced.

**Conclusion:**

In KOA patients with tibial malalignment, the CORA is predominantly located in the diaphysis rather than in the proximal metaphysis, where osteotomies are typically performed. This discrepancy leads to iatrogenic translational deformities. Future research should investigate the clinical impact of these deformities to optimize osteotomy planning and potentially improve long-term surgical outcomes.

## Introduction

Osteoarthritis (OA) affected 595 million people globally in 2019,^1^ with knee osteoarthritis (KOA) being the most common type.^2^ As obesity and life expectancy rise,^1,3-6^ KOA prevalence increases.^2^ Lower limb malalignment is a risk factor for KOA progression,^7-9^ shifting the knee’s mechanical axis and accelerating cartilage degeneration.^8-11^ Realignment osteotomies are a well-established treatment for younger patients to postpone knee arthroplasty,^12-14^ which is important as they have a higher risk of revision surgery.^15^

Preoperative planning for knee osteotomy is essential for optimal outcomes.^16^ Early methods like the Fujisawa point, Miniaci line, and Dugdale method,^17^ have evolved into the current planning nomenclature proposed by Paley.^18^ Modern planning methods focus on calculating the desired wedge height for knee osteotomies, with corrections typically performed at the tibial and femoral metaphyseal ends.^17,19,20^ Interestingly, Paley’s^18^ approach extends beyond this conventional method by emphasizing the correction of deformities at their origin, a concept well-known in reconstructive surgery.

Building on this concept, the center of rotation angulation (CORA) is the point where the mechanical axes of a deformed bone intersect.^18,21^ By correcting deformities at the CORA, the risk of introducing secondary translational deformities is minimized.^18^ Tibial osteotomies for unicompartmental KOA are preferably performed at the proximal tibial end, as this region is characterized by superior bone healing due to its high trabecular density and vascularization^22-24^ compared with the mid-diaphysis. Consequently, in these procedures, potential translation of the bone is often assumed and remains unaddressed. Therefore, our study aims to identify the CORA of patients diagnosed with KOA and associated tibial malalignment. This will provide crucial insights into the potential occurrence of iatrogenic deformities.

## Methods

### Patients

In the prospective Applied Public-Private Research enabling OsteoArthritis Clinical Headway (IMI-APPROACH) cohort, 297 KOA participants from 5 European centers were included.^25-29^ Some of these participants also exhibited malalignment of the femur or tibia. Detailed inclusion and exclusion criteria have been previously published.^30^ The study was approved by Institutional Review Boards, in accordance with all relevant ethical and legal regulations. The study was registered under clinicaltrials.gov number: NCT03883568, and informed consent was obtained from all participants.

### Imaging Assessment

All patients underwent low-dose whole-body computed tomography (CT) scans. The tibia and fibula were segmented from the CT scans using validated software (Mimics; Materialise, Leuven, Belgium). Bone geometry analyses were performed in 3-matic (Materialise). The analyses involved a semi-automated method as the functions were scripted in Python language (3-match plugin).

#### 3D tibial coordinate system

A patient-specific 3D coordinate system was constructed per tibiae. The mechanical axis ran from the tibial eminences to the distal tibial plafond. The transversal plane had its origin at the tibial eminences, with the mechanical axis as normal vector. The sagittal plane, perpendicular to the transversal plane, crossed the posterior cruciate ligament attachment and medial tuberosity border (Akagi’s line).^31^ The coronal plane, perpendicular to both transversal and sagittal planes, originated at the tibial eminences.

#### CORA calculation

**Figure 1.**
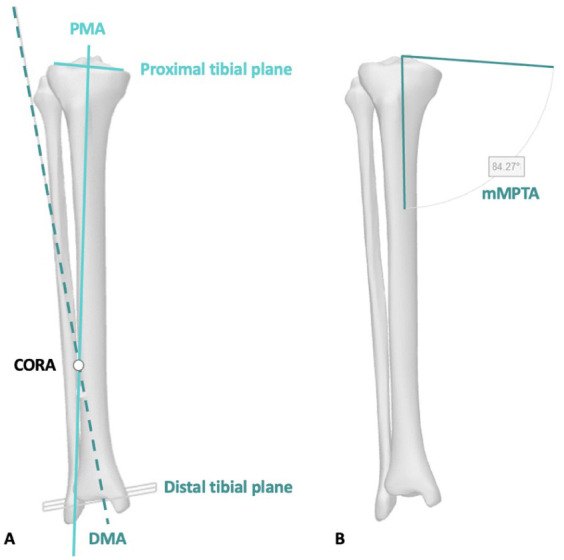
Definition of the CORA and the mMPTA. (**A**) The CORA (center of rotation angulation) is the intersection of the proximal mechanical axis (PMA, black dotted line) and the distal mechanical axis (DMA, gray line) of the tibia. (**B**) The mMPTA (mechanical medial proximal tibial angle) is the angle formed between the mechanical axis of the tibia and the tangent of the proximal tibial plateau on the coronal plane.

The method fitted a plane to the articulating surface of the medial and lateral tibial plateaus (proximal tibial plane) and projected a line distally originating from the center of the eminence spines at an angle of 87° (coronal view) relative to the proximal tibial plane (proximal mechanical axis (PMA)) (**Fig. 1A**). In addition, a plane was fitted to the articulating surface of the distal tibial plafond (distal tibial plane), and a line was projected proximally at an 89° angle (coronal view) relative to the distal tibial plane (distal mechanical axis (DMA)), originating from the center of the distal tibial plafond (**Fig. 1A**). The crossing of the two lines represented the CORA of the coronal tibial deformity, and the location of this CORA was calculated with respect to the tibial eminence center (knee joint center) (**Fig. 1A**).

#### Bone deformity

Coronal bone deformities of the tibia, including the mechanical medial proximal tibial angle (mMPTA), was assessed using Paley’s^18^ method. The mMPTA was defined as the angle (coronal view) between the mechanical axis of the tibia and the tangent tot the proximal tibial plateau (**Fig. 1B**). Neutral mMPTA ranged from 85° to 90°. A varus mMPTA was defined as <85°, while a valgus mMPTA was >90°.^18^ A total of 30 tibiae were included: 10 with a neutral mMPTA, 10 with a varus mMPTA, and 10 with a valgus mMPTA. Participants with tibial deformity were randomly selected to ensure a representative distribution of the mMPTA across the different alignment groups (neutral, varus, and valgus).

### The Concept of Secondary Translational Deformities in Osteotomies

**Figure 2.**
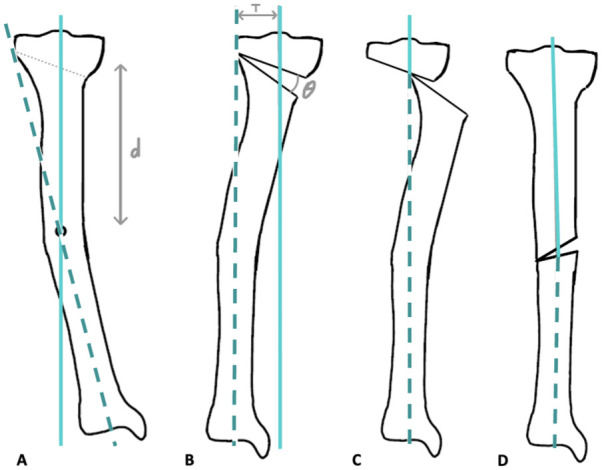
The concept of iatrogenic translational deformities in osteotomies when the osteotomy is performed outside the CORA. The PMA is shown in red, the DMA in blue, and the CORA is marked by the green circle. (**A**) The tibia with deformity. (**B**) High tibial osteotomy performed away from the CORA leads to parallel PMA and DMA. (**C**) Translation is required to realign the PMA and DMA. (**D**) No translation is necessary when the osteotomy is performed at the CORA.

In osteotomies, translation refers to the sideward displacement of the distal segment of the tibia. This occurs when the osteotomy is not performed at the CORA, which is the optimal location for performing an osteotomy without creating a secondary deformity.^21^ The reason for this translation lies in the kinematics of the correction. A bone deformity creates an angular malalignment, and when an osteotomy is performed at a distance from the CORA, the bone must be corrected by rotating or angulating the distal segment to realign it with the proximal segment. This causes a sideward shift in the direction of the angular correction. Stated otherwise, when an osteotomy is performed outside the CORA, angular correction alone can render the PMA and DMA parallel while still malaligned, necessitating medial translation of the distal segment for complete alignment (see **Figure 2**).

Translation is influenced by 2 factors: the distance from the CORA to the osteotomy site^32^ and the correction angle.^32^ Greater distance and larger correction angles increase translational displacement. The translation can be calculated by

(1) T=d×tan(θ)

In this equation, T refers to the sideward displacement of the distal segment, d is the distance from the CORA to the osteotomy site, and θ is the correction angle (degrees) (**Fig. 2**). For a varus tibia, the correction aimed to realign the mMPTA to 90°, whereas for a valgus tibia, the correction targeted an mMPTA of 85°. This aligns with clinical practice, where we apply slight overcorrection in KOA patients.^33^

### CORA Location

In addition to assessing the magnitude of the secondary deformity, the location of the CORA was also evaluated. The location of the CORA on the tibia was defined as a percentage of the tibial length, with 100% representing the proximal end and 0% representing the distal end. This method allowed for a descriptive analysis of CORA positions in tibiae with varus and valgus deformities. By comparing these positions, differences in CORA location between the 2 deformity groups were identified.

### Statistical Analysis

All statistical analyses were conducted using Statistical Package for the Social Sciences (SPSS) Version 29.0 software. Descriptive statistics were computed, including means and standard deviations (SD) for continuous variables, and numbers and percentages for categorical data.

## Results

### Locations of CORA in the Tibia

A total of 30 tibiae were included in this study, consisting of 10 individuals with varus alignment of the tibiae, 10 with neutral alignment of the tibiae, and 10 with valgus alignment, based on the mMPTA, all of whom presented with early-stage KOA. Most patients were female (73%), and the mean age was 62.9 ± 8.0 years. The mean mMPTA was 87.3° ± 3.3°.

**Figure 3.**
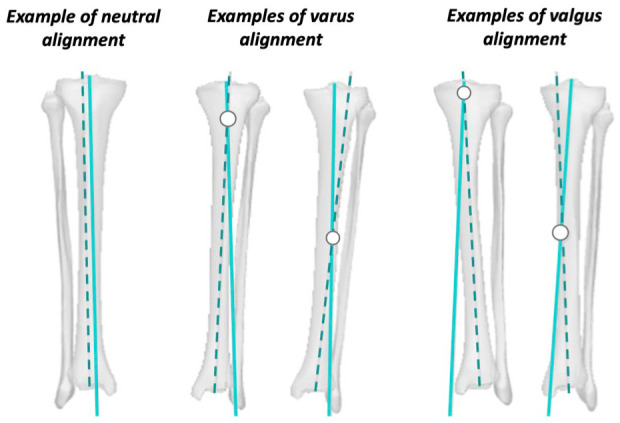
Examples of CORA determinations in different tibiae. (**A**) Neutral tibia, where the PMA (solid line) and DMA (dotted line) run nearly parallel, indicating no CORA. (**B**) Varus tibia with an example where the CORA is in both the metaphyseal and diaphyseal regions. (**C**) Varus tibia with another example of the CORA located in both the metaphyseal and diaphyseal regions.

In neutral tibial alignment, the mean mMPTA was 87.5° ± 1.4°. Since there was no deformity in these bones, the PMA and DMA (**Fig. 1**) were nearly parallel, and no CORA was present (**Fig. 3A**). In varus alignment, the mean mMPTA was 83.4° ± 1.0°. The CORA in the varus group was located within the proximal metaphyseal region in 1 case and within the diaphyseal region in 9 cases (**Fig. 3B**). In the valgus alignment group, the mean mMPTA was 91.0° ± 0.7°. The CORA of the valgus group was located within the metaphyseal region in 2 cases and within the diaphyseal region in 8 cases (**Fig. 3C**).

**Figure 4.**
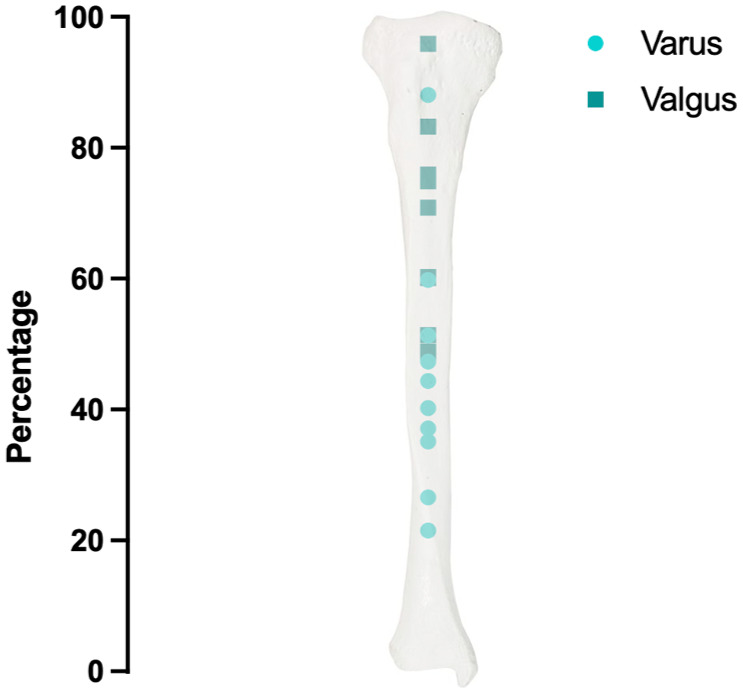
CORA locations relative to tibial length, presented separately for varus and valgus tibiae, with 100% representing the proximal end and 0% representing the distal end.

In the varus alignment group, the mean CORA was located at 54.9 ± 18.8% of the tibial length, ranging from 11.9 to 78.5% (**Fig. 4**). In the valgus alignment group, the mean CORA was situated at 33.0 ± 15.9% of the tibial length, with a range from 4.1 to 51.6% (**Fig. 4**). In the varus group, the CORA was located more distally than in the valgus group (**Fig. 4**).

### Secondary Translational Deformities in Osteotomies

**Figure 5.**
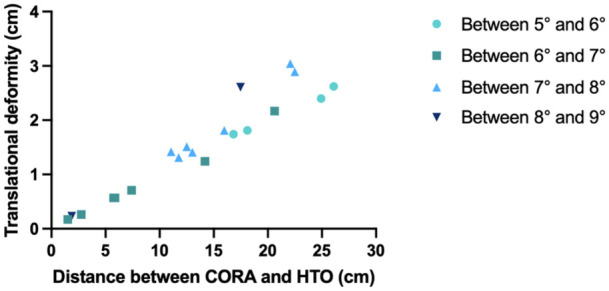
Secondary translation deformity values are plotted against the distances measured between the CORA and the high tibial osteotomy plane. Distinctions were made for different correction angles, highlighting the relationship between the secondary translation and the correction magnitude.

For an angular correction of the varus mMPTA to 90°, the mean translation when performing the osteotomy on a varus tibia at the metaphysis, as opposed to at the CORA site, was 2.09 cm ± 0.85 cm, with a range from 0.17 cm to 3.04 cm. In contrast, for an angular correction of the mMPTA to 85° when correcting a valgus tibia, the mean translation was 0.96 cm ± 0.56 cm, with a range from 0.23 cm to 1.81 cm. **Figure 5** illustrates the relationship between the distances from the CORA to the high tibial osteotomy plane and the corresponding calculated secondary translational deformity.

## Discussion

This study aimed to identify the CORA in KOA patients with tibial malalignment and explore secondary translation in tibial osteotomies not performed at the CORA, all conducted in a cohort comprising patients with KOA. A key finding was that 85% of the CORAs were in the diaphysis, leading to secondary translational deformities of up to 3 cm, a novel finding not previously reported in the knee osteotomy literature. Identifying and targeting the CORA during osteotomy planning may improve knee realignment and postoperative outcomes.

The clinical impact of these secondary translational deformities is unclear, as no studies have focused on the effect of CORA locations in KOA patients with tibial malalignment. Potential consequences may include altered gait mechanics, increased joint wear, and unfavorable force distribution within the affected compartment; however, these hypotheses require further investigation. Most existing research on osteotomies in KOA focuses on the mechanical hip-knee-ankle angle.^9,34,35^ What we do know is that osteotomy is an effective treatment for relatively young KOA patients, as it can delay the need for knee arthroplasty by over 10 years.^12,13,36,37^ Predictors for an increased likelihood of conversion to arthroplasty include radiographic OA severity, pain, female sex, age, and body mass index (BMI), with radiographic OA severity being the strongest predictor.^36,38^ While osteotomies are effective, improving long-term outcomes is crucial, especially as KOA increases in prevalence.^2^ Optimizing osteotomy techniques could improve knee function and patient satisfaction, but further research is needed to validate these findings and assess their impact on clinical outcomes and the long-term effectiveness of osteotomies in KOA treatment.

In addition to the 30 tibiae analyzed, 10 extra tibiae exhibited translational deformities (S-shaped) without prior osteotomy (Appendix). These deformities can be conceptualized as a displacement in which the distal segment shifts relative to the proximal segment,^18,21^ which is also observed when an osteotomy is not performed at the CORA. In valgus mMPTA patients, the deformities show a varus mechanical lateral distal tibial angle. In such cases, 2 osteotomies with opposing corrections at each level are recommended.^21^ These translational deformities, common in pediatric patients,^39-41^ can cause abnormal gait, joint wear, and premature OA.^41^ To prevent these long-term complications, corrective osteotomies are often performed in children to align the bones properly.

Historically, osteotomies are performed in the metaphysis^17,42^ due to better bone healing compared with the diaphysis.^22-24^ The diaphysis consists primarily of dense cortical bone with less robust intraosseous blood supply, whereas the metaphysis is characterized by more metabolically active and vascular trabecular bone.^22^ This difference likely contributes to higher rates of nonunion and hinge fractures in diaphyseal osteotomies.^23,24^ Despite the risk of translation, the benefits of fracture healing often outweigh this concern. In practice, secondary translation is often overlooked, as the mechanical axis is shifted, which is the primary goal of the procedure.

The concept of secondary translational deformity has been described in the literature,^18,21,32,41^ but no studies have focused on CORA location in KOA patients with tibial malalignment. Barksfield and Monsell^32^ concluded that translational deformities can be predicted by the angular correction and distance from the CORA. However, their study did not focus on KOA patients and involved smaller simulated distances. Our study shows that these distances can reach up to 3 cm, leading to larger translational deformities that require further investigation—although this was shown in a relatively small sample size. Therefore, future research is needed to evaluate CORA locations in a larger cohort of KOA patients with lower limb malalignment who are candidates for osteotomy. Future research should also assess the long-term effects of these deformities on knee function, survival rates, and patient outcomes. Moreover, the influence of these deformities on force distribution in the affected compartment should be explored, and whether performing osteotomies away from the CORA enhances or diminishes this effect.

High tibial osteotomy is indicated primarily to correct varus or valgus malalignment in unicompartmental KOA.^43^ Feucht *et al.*^44^ demonstrated that mild varus malalignment often results from a deformity in the joint line convergence angle (JLCA), not the bones themselves. In these cases, correcting the JLCA along with tibial realignment is recommended. Osteotomy in the proximal tibia is advantageous for JLCA deformities, but whether osteotomy is the right approach for JLCA deformities alone remains unclear. In clinical practice, during a high tibial osteotomy, correction of the JLCA is performed alongside tibial realignment. For patients undergoing osteotomy without bone deformities but with a deformity of the JLCA, performing the osteotomy in the proximal tibia is advantageous, as the CORA would lie within the knee joint for these patients. However, the question remains whether osteotomy is the appropriate indication for patients with only a deformity of the JLCA.^38^ Further research is needed to better understand the optimal approach for patients with JLCA deformities.

Several limitations of this study should be acknowledged. The study included 30 tibiae, a small sample size, with the goal of raising awareness among orthopedic surgeons that the CORA in tibial malalignment is typically not always located in the proximal metaphysis. In addition, the mean age of our cohort was higher than the typical age at which high tibial osteotomy is performed, which may affect the generalizability of our findings. Our study focused solely on patients with tibial malalignment. Future research should also examine its potential impact on femoral double-level osteotomies. Second, CORA determination was based on CT imaging, not whole-leg radiography, which remains the clinical gold standard. However, CT scans eliminate positioning factors,^45^ and Roth *et al.*^46^ found minimal differences between weight-bearing and non-weight-bearing conditions for bony alignment. Third, in coronal alignment osteotomies for KOA patients, the osteotomy extends to the hinge point. Consequently, operating at the CORA inevitably induces minimal secondary translational deformity, as the center of rotation does not coincide with the CORA. Finally, the standardized correction angles used in this study may differ from individualized surgical planning, which could lead to different magnitudes of translational shifts and alignment outcomes. In clinical practice, surgeons increasingly avoid over-corrections, instead aiming for a more neutral alignment. Moreover, our analysis focused on secondary translation following a tibial osteotomy, whereas larger corrections in current practice are often managed with double-level osteotomies, which distribute the correction across two bones and therefore reduce the degree of secondary translation.

## Conclusion

This study identified the CORA in patients with KOA and tibial malalignment, highlighting the secondary translational deformities that arise because of not performing the osteotomy at the CORA. We concluded that 85% of CORAs were in the diaphysis and not located in the proximal metaphysis, leading to secondary translational deformities of up to 3 cm after high tibial osteotomy. Future studies should focus on the clinical implications to possibly improve both its effectiveness and long-term sustainability of osteotomies.

## Figures and Tables

**Figure A1.**
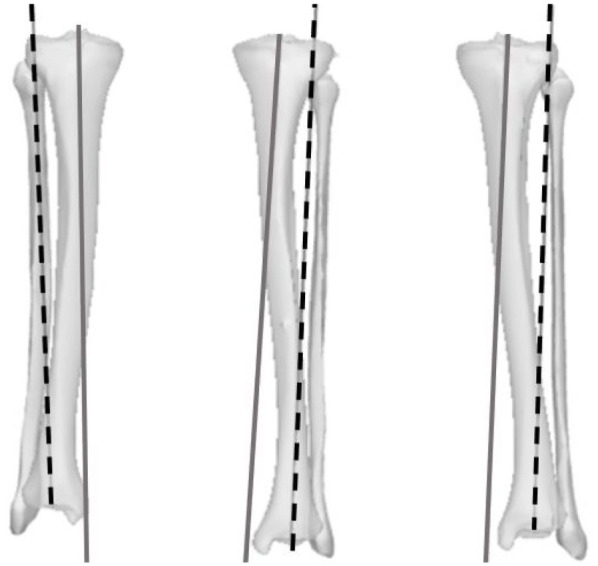
Examples of the translational deformities in IMI-APPROACH, where the PMA and DMA run nearly parallel. The mean mMPTA and mLDTA were 91.0 ± 0.4 and 93.9 ± 1.6, respectively.
